# Ceftriaxone Treatment Weakens Long-Term Synaptic Potentiation in the Hippocampus of Young Rats

**DOI:** 10.3390/ijms22168417

**Published:** 2021-08-05

**Authors:** Tatyana Y. Postnikova, Sergey L. Malkin, Maria V. Zakharova, Ilya V. Smolensky, Olga E. Zubareva, Aleksey V. Zaitsev

**Affiliations:** Sechenov Institute of Evolutionary Physiology and Biochemistry of RAS, 44, Toreza Prospekt, 194223 Saint Petersburg, Russia; tapost2@mail.ru (T.Y.P.); adresatt@gmail.com (S.L.M.); zaharova-masha@yandex.ru (M.V.Z.); smolensky.ilya@gmail.com (I.V.S.); zubarevaoe@mail.ru (O.E.Z.)

**Keywords:** long-term potentiation, NMDA receptor, hippocampus, EAAT2, memory

## Abstract

Disrupted glutamate clearance in the synaptic cleft leads to synaptic dysfunction and neurological diseases. Decreased glutamate removal from the synaptic cleft is known to cause excitotoxicity. Data on the physiological effects of increased glutamate clearance are contradictory. This study investigated the consequences of ceftriaxone (CTX), an enhancer of glutamate transporter 1 expression, treatment on long-term synaptic potentiation (LTP) in the hippocampus of young rats. In this study, 5-day administration of CTX (200 mg/kg) significantly weakened LTP in CA3-CA1 synapses. As shown by electrophysiological recordings, LTP attenuation was associated with weakening of N-Methyl-D-aspartate receptor (NMDAR)-dependent signaling in synapses. However, PCR analysis did not show downregulation of NMDAR subunits or changes in the expression of α-amino-3-hydroxy-5-methyl-4-isoxazolepropionic acid receptor (AMPAR) subunits. We assume that extracellular burst stimulation activates fewer synapses in CTX-treated animals because increased glutamate reuptake results in reduced spillover, and neighboring synapses do not participate in neurotransmission. Attenuation of LTP was not accompanied by noticeable behavioral changes in the CTX group, with no behavioral abnormalities observed in the open field test or Morris water maze test. Thus, our experiments show that increased glutamate clearance can impair long-term synaptic plasticity and that this phenomenon can be considered a potential side effect of CTX treatment.

## 1. Introduction

Glutamate is a primary excitatory neurotransmitter in the central nervous system, acting through ionotropic and metabotropic receptors [1,2]. Ionotropic α-amino-3-hydroxy-5-methyl-4-isoxazolepropionic acid receptors (AMPARs) play a major role in driving rapid depolarization of postsynaptic neurons and action potential firing. N-methyl-D-aspartate receptors (NMDARs) are thought to be very important for controlling synaptic plasticity [3]. The intensity and duration of excitatory synaptic activity play an essential role in information exchange between neurons, with these processes critically dependent on how long glutamate remains in the synaptic cleft [4].

Under physiological conditions, the extracellular glutamate concentration is maintained at a range of 0.6–2.0 µM [5]. However, in many pathological conditions associated with impaired glutamate clearance, the glutamate concentration in the synaptic cleft accumulates over time, with excessive extracellular glutamate causing excitotoxicity in some cases [4].

The molecular machinery of glutamate clearance in the cerebral cortex and hippocampus mainly involves three excitatory amino acid transporter (EAAT) proteins: EAAT1 (glutamate aspartate transporter [GLAST], murine equivalent), EAAT2 (glutamate transporter 1 [GLT1]), and EAAT3 (excitatory amino acid carrier 1 [EAAC1]) [5]. GLAST and GLT1 are expressed primarily in astrocytes but may be expressed also in other cell types [6,7]. EAAC1 is expressed in neurons only, mostly in postsynaptic membranes [8]. GLT1 transports more than 90% of synaptic glutamate in the hippocampus, and GLT1 accounts for 80% of EAAT molecules [9,10]. Therefore, enhancing GLT1 expression with pharmacological and genetic methods is considered a promising therapeutic option for diseases associated with impaired glutamate transport [5,10,11].

Currently, ceftriaxone (CTX), a cephalosporin antibiotic, is one of the most studied GLT1 expression enhancers [12,13,14]. CTX causes both acute and long-term increases in GLT1 expression, and it also enhances the functional activity of GLT1 [13]. In our previous study, CTX administration had mild anticonvulsant effects on pentylenetetrazole (PTZ)-induced convulsions and in a maximal electroshock threshold test [14]. Other studies revealed that CTX reduced signs of kindling in the PTZ model [15,16]. In addition to anticonvulsive effects, studies have demonstrated that CTX has neuroprotective action [10,13,17] and anti-ischemia effects [18].

It can be assumed that enhanced GLT1 expression in a normal brain will increase glutamate uptake from the synaptic cleft and potentially affect excitatory transmission in neuronal networks and synaptic plasticity. Experimental data in this area are limited and somewhat contradictory. In a previous study, we detected no significant change in the efficacy of glutamatergic synaptic neurotransmission in the hippocampus and temporal cortex following CTX treatment. However, we detected attenuation of field excitatory postsynaptic potential (fEPSP) amplitudes in CA1 evoked by intense electrical stimulation of Schaffer collaterals in the CTX-treated group [14]. Other laboratories have shown that CTX-treated rats display significant impairment in novel object recognition, which is consistent with a hippocampal deficit [19]. Upregulation of the glutamate transporter GLT1 by CTX severely impaired mGluR-dependent long-term depression (LTD) induced in rat mossy fiber (MF)–CA3 synapses by repetitive stimulation of afferent fibers. Furthermore, CTX-induced GLT1 upregulation significantly reduced the magnitude of LTP at MF–CA3 synapses but not at Schaffer collateral–CA1 synapses [20].

Therefore, in this study, we aimed to investigate the characteristics of synaptic plasticity in the hippocampus of young rats after CTX administration.

## 2. Results

### 2.1. LTP Was Attenuated in the CA1 Hippocampal Area after CTX Treatment

**Figure 1:**
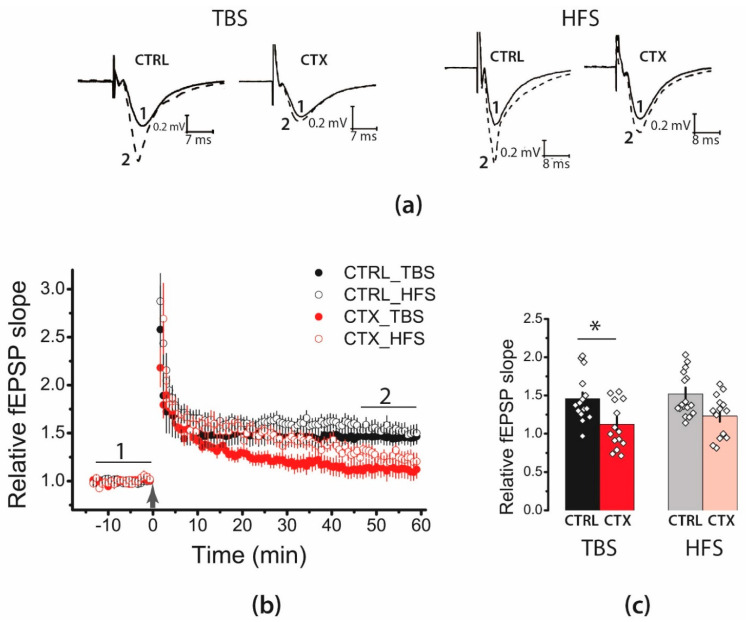
Long-term potentiation (LTP) was attenuated in the CA1 hippocampal area after administration of ceftriaxone (CTX). (**a**) Representative examples of field excitatory postsynaptic potential (fEPSP) before induction (1) and 60 min after theta-burst stimulation (TBS) or high-frequency stimulation (HFS) (2). (**b**) Diagram showing changes in the value of the normalized slope of fEPSP in the control (CTRL) and CTX-treated (CTX) animals after TBS or HFS. Black arrow indicates the moment of LTP induction (TBS or HFS). (**c**) Diagram illustrating the differences in LTP between the control and CTX-treated (CTX) animals according to the type of induction protocol. All the data are presented as mean ± standard error of the mean. * *p* < 0.05: a significant difference versus the control group (’Tukey’s post hoc test).

As overexpression of GLT1 may affect synaptic plasticity due to changes in glutamate dynamics in synaptic clefts [20], we investigated whether CTX treatment for 5 days affects synaptic plasticity in hippocampal Schaffer collateral-CA1 synapses (Figure 1). We used two protocols for LTP induction: theta-burst stimulation (TBS) and high-frequency stimulation (HFS). In the control animals, both protocols induced substantial LTP (TBS: 1.46 ± 0.08, *n* = 15; HFS: 1.51 ± 0.08, *n* = 17). LTP was attenuated in the CTX-treated groups as compared with that in the control groups (two-way analysis of variance [ANOVA], effect of CTX treatment: F_1,56_ = 13.8; *p* < 0.001; TBS: 1.12 ± 0.09, *n* = 14; HFS: 1.23 ± 0.07, *n* = 14). We found no differences in the efficacies of the two LTP induction protocols (HFS vs. TBS: F_1,56_ = 1.03; *p* = 0.31) and no interaction effect (type of LTP induction protocol × CTX treatment: F_1,56_ = 0.08; *p* = 0.77).

The initial phase of LTP (5–15 min) in the treated animals was almost the same as that recorded in the controls. Subsequently, synaptic potentiation declined in the CTX-treated group. Thus, LTP maintenance was impaired after the CTX treatment, regardless of the type of induction protocol used.

**Figure 2:**
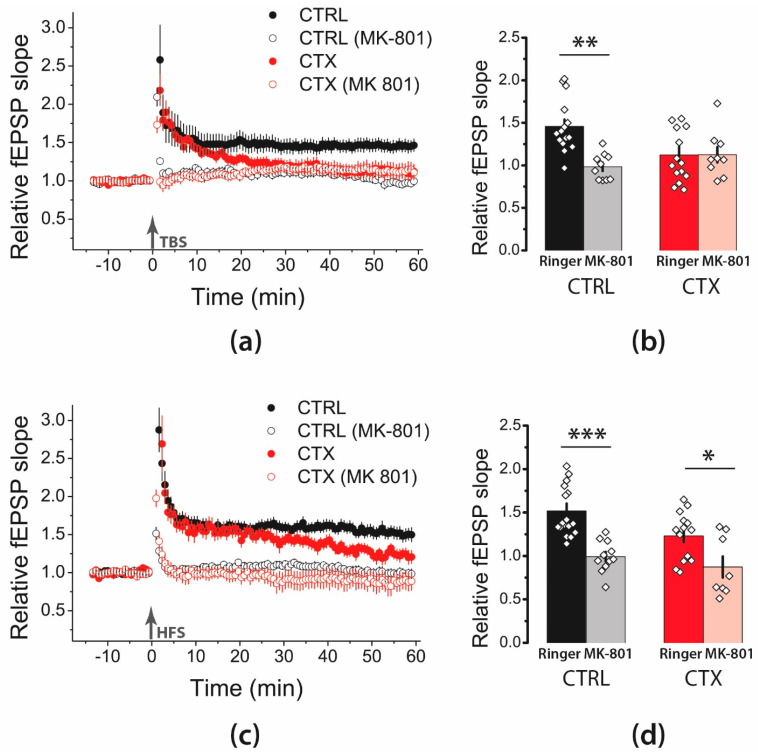
NMDAR-dependent mechanism of LTP induction is unaltered following administration of CTX. (**a**,**c**) Diagram showing changes in the value of the normalized slope of fEPSP in the control (CTRL) and CTX-treated groups (CTX) after TBS (**a**) and HFS (**c**). (**b**,**d**) Diagrams illustrating the magnitude of plasticity in the control and CTX groups in the presence of MK-801, after TBS (**b**) or HFS (**d**). Black arrow indicates the moment of LTP induction (TBS or HFS). Two-way ANOVA following Tukey post hoc tests were used. All the data are presented as mean ± standard error of the mean. * *p* < 0.05, ** *p* < 0.01, *** *p* < 0.001: A significant difference versus the control group (Tukey’s post hoc test).

Many studies have demonstrated that LTP in the hippocampal CA1 region is an NMDAR-dependent process [21,22]. However, in some pathological conditions, LTP induction in the hippocampus may become NMDA-independent [23]. To test whether LTP induction in CA3-CA1 synapses following CTX treatment was NMDAR-dependent, we induced LTP in the presence of the NMDAR channel blocker MK-801 (10 μM). The application of MK-801 significantly affected LTP induction following the TBS protocol (Figure 2a,b; two-way ANOVA, effect of MK-801: F_1,44_ = 7.3, *p* < 0.01) and HFS protocol (Figure 2c,d; F_1,46_ = 26, *p* < 0.001). In the control groups, the MK-801 application markedly inhibited LTP induction in both the TBS (Figure 2a,b; 0.98 ± 0.05, *n* = 10) and HFS protocol groups (Figure 2c,d; 0.99 ± 0.05, *n* = 11). In the CTX-treated animals, the effect of MK-801 was significant only in the HFS protocol group; no LTP was induced in the presence of MK-801 (HFS, MK-801: 0.87 ± 0.12; *n* = 8). In the TBS protocol group, in which LTP was almost absent, MK-801 did not affect synaptic plasticity (TBS, MK-801: 1.13 ± 0.09; *n* = 9). Thus, these data suggested that LTP induction in the control and CTX-treated animals is an NMDAR-dependent process.

### 2.2. NMDA-Mediated Signaling during Train Stimulation Was Attenuated after CTX Treatment

**Figure 3:**
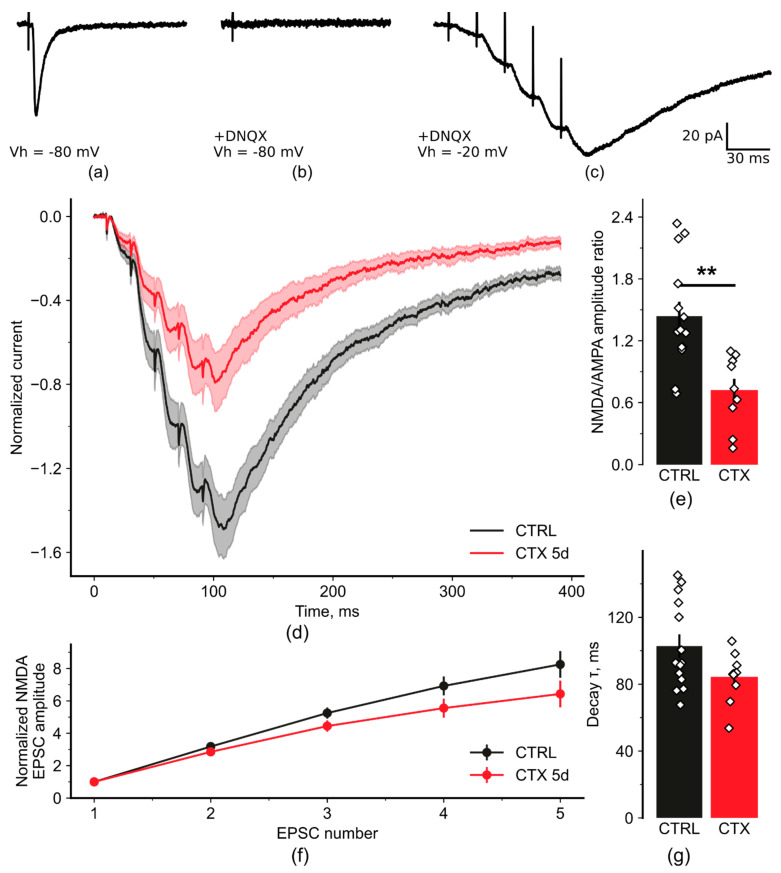
The N-methyl-D-aspartate receptor (NMDAR)/α-amino-3-hydroxy-5-methyl-4-isoxazolepropionic acid receptor (AMPAR) ratio was significantly reduced in the CTX-treated animals, with no effect on NMDAR-mediated current kinetics. (**a**) AMPAR-mediated evoked excitatory postsynaptic currents (eEPSCs) recorded at −80 mV in the presence of GABAaR and GABAbR antagonists bicuculline (10 µM) and CGP 55845 (5 µM). (**b**) Complete block of the AMPAR-mediated current by bath-applied DNQX (20 µM). (**c**) NMDAR-mediated eEPSCs recorded in the same neuron in the presence of DNQX at −20 mV with the same stimulus current amplitude. (**d**) Average NMDAR-mediated currents normalized by the AMPAR-mediated eEPSC amplitude for each group. The transparent areas represent the standard error of the mean for each trace. (**e**) NMDA/AMPA ratios measured as the peak NMDAR-mediated current amplitude (for the last EPSC) divided by the peak AMPAR-mediated current amplitude. The asterisk represents a significant difference according to the Student’s *t*-test (*p* < 0.01). (**f**) Average NMDAR-mediated peak eEPSC amplitudes normalized by the first EPSC amplitude for each recorded cell. (**g**) Decay τ measured at the 80–20% amplitude of the last NMDAR-mediated EPSC in the train using a monoexponential approximation. ** *p* < 0.01, a significant difference versus the control group according to the Student’s *t*-test.

LTP attenuation might be caused by insufficient activation of NMDARs during the induction protocol. To test this possibility, we investigated the NMDAR-mediated responses of CA3-CA1 synapses during a TBS-like protocol in the control and CTX groups using whole-cell recordings. First, we estimated the relative impact of NMDAR- and AMPAR-mediated currents in evoked excitatory postsynaptic currents (eEPSCs). We recorded AMPAR-mediated eEPSCs in CA1 pyramidal neurons in the presence of the GABAaR and GABAbR antagonists bicuculline (10 µM) and CGP 55845 (5 µM), respectively, clamping the membrane potential at −80 mV, which eliminated NMDAR-mediated currents due to the Mg^2+^ block. We adjusted the stimulus amplitude for each cell to elicit a clear monosynaptic AMPAR-mediated EPSC with an amplitude of 100–200 pA, which is in the subthreshold range for CA1 neurons (Figure 3a) and is similar to that used in the LTP experiments. After recording 15 EPSCs, we then applied a selective AMPAR antagonist DNQX (20 µM) and continued the stimulation for 10 min to ensure a complete block of the EPSCs (Figure 3b). We then recorded NMDAR-mediated EPCSs at −20 mV holding potential. Finally, to mimic TBS, we applied trains of five stimuli at 50 Hz of the same strength and duration as for the AMPAR-mediated eEPSCs (Figure 3c).

We calculated NMDAR/AMPAR ratios by dividing the peak NMDAR-mediated amplitude for the last EPSC in the train by the peak of the AMPAR-mediated EPSC amplitude. We found that the NMDAR/AMPAR ratio in the CTX group was significantly lower that that in the control group (Figure 3e; control: 1.40 ± 0.14, *n* = 14; CTX: 0.70 ± 0.12, *n* = 9; Student’s *t*-test = 3.65, *p* < 0.01). Thus, similar synaptic stimulation, as estimated by AMPAR-mediated currents, results in much lower NMDAR-mediated conductance during train stimulation (Figure 3d) and consequently less transfer of calcium ions to postsynaptic neurons. These results explain the decrease in the LTP magnitude in the CTX group.

Next, we analyzed other properties of NMDAR-mediated EPSCs that may affect the amplitude of the evoked response. To assess summation properties, we measured the peak amplitudes of each consecutive eEPSC and normalized them to the first eEPSC amplitude. We compared the resulting normalized amplitudes in the control and CTX groups using a one-way repeated-measures ANOVA and found no difference between these groups (Figure 3f; F_3,63_ = 2.2, *p* = 0.10).

We then measured the decay kinetics of the NMDAR-mediated EPSCs by fitting the 80-20% decay phase of the train’s last response with a single exponential function. There was no significant difference in the decay kinetics between the control and CTX groups. Nevertheless, there was a tendency toward a decrease in tau decay (Figure 3g; control: 103 ± 7 ms, *n* = 14; CTX: 84 ± 5 ms, *n* = 9; Student’s *t*-test = 1.9, *p* = 0.067). Therefore, neither the summation nor time course of NMDA EPSCs was affected in the CTX group.

### 2.3. Expression of Glutamate Receptor Subunit Genes Encoding the NMDAR following CTX Treatment

**Figure 4:**
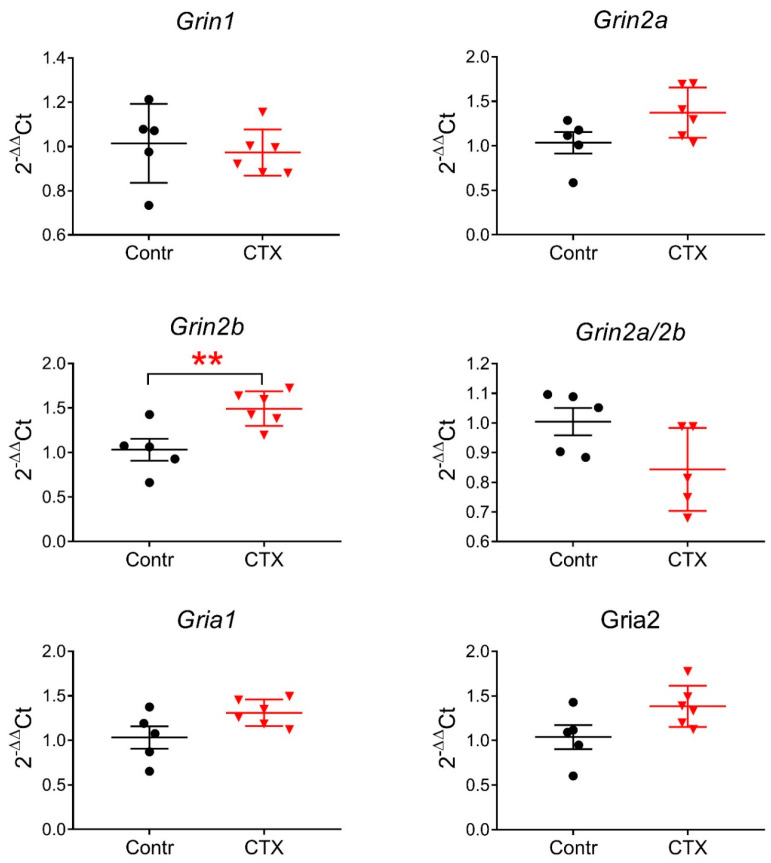
The relative expression of the *Grin1*, *Grin2a*, *Grin2b*, *Gria1*, *Gria2* genes, and *Grin2a*/*Grin2b* ratio in the dorsal hippocampal area after CTX treatment. Contr—control group, CTX—experimental group. Data are presented as a mean with a standard error of the mean. Each dot (circle, triangle) represents a value obtained in individual animal. ** *p* < 0.01, a significant difference versus the control group according to the Student’s *t*-test.

Smaller NMDAR-mediated synaptic responses might be explained by downregulation of NMDAR and/or upregulation of AMPAR expression following CTX treatment. Therefore, we compared the expression of glutamate receptor subunit genes encoding the NMDAR (*Grin1*, *Grin2a*, and *Grin2b*) and AMPAR (*Gria1*, *Gria2*) in the dorsal hippocampus (Figure 4). No changes in the production of mRNA Grin1, an obligate NMDAR subunit, were detected, suggesting that the total amount of the NMDARs, most likely, did not change. The expression of the *Grin2b* gene increased in the dorsal hippocampus (*t* = 3.25; *p* < 0.01) but not the expression level of *Grin2a* or the *Grin2b*/*Grin2a* expression ratio. Together, these findings exclude the hypothesis of downregulation of NMDAR expression in the hippocampus after CTX administration.

We also detected no significant changes in the expression of AMPAR subunits, as the expression levels of *Gria1* and *Gria2* were similar in the control and CTX groups (Figure 4). Therefore, the hypothesis of upregulation of AMPARs was also excluded.

### 2.4. Rats’ Behavior Is Not Impaired after Ceftriaxone Treatment

#### 2.4.1. Open Field Test

**Figure 5:**
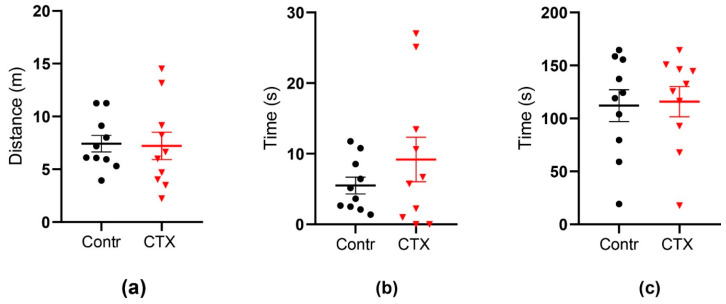
The behavior of CTX-treated rats in the open field test does not change. (**a**) Distance traveled by a rat during the testing time, (**b**) the time rat spent in the center of the arena, (**c**) the time rat spent in thigmotaxis. Data are presented as a mean with a standard error of the mean. Each dot (circle, triangle) represents a value obtained in individual animal.

We detected no changes in rat behavior following the CTX treatment (Figure 5). The rats in the control and CTX-treated groups traveled the same distance in the arena (control: 7.4 ± 0.8 m CTX: 7.2 ± 1.3 m) and spent a similar time in the center (5.5 ± 1.2 and 9.2 ± 3.2 s, respectively). Thigmotaxis was similar in the two groups (112 ± 15 and 116 ± 14 s, respectively). Thus, the CTX treatment impaired neither the explorative activity nor anxiety levels of the rats.

#### 2.4.2. Morris Water Maze

**Figure 6:**
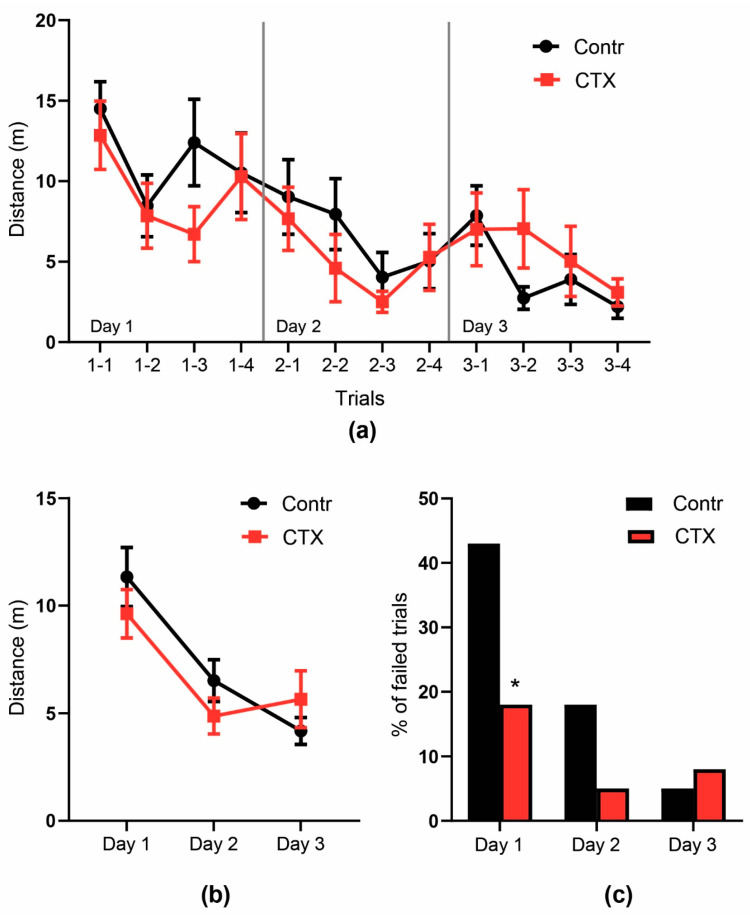
The behavior of CTX-treated rats in the Morris water maze. (**a**) distance traveled from the start point to the hidden platform during the 90-s trial, (**b**) the rate of failed trials during each day, * significant differences between the groups, Fisher’s exact test, *p* < 0.05, (**c**) mean distance traveled during four trials each day.

In the Morris water maze, there was no difference in short- or long-term memory of the rats in the CTX and control groups, as the dynamics of finding the hidden platform were the same in both groups (Figure 6). Short-term memory was estimated by the distance traveled to reach the hidden platform in four trials on Day 1 of training. A repeated-measures ANOVA revealed no significant differences in the dynamics of the control and CTX-treated groups in the distance traveled to reach the hidden platform (F_3,14_ = 0.78, *p* = 0.52, Figure 6a). However, the CTX-treated rats had significantly fewer failed trials on Day 1 (18% vs. 43%, *p* < 0.05, ’Fisher’s exact test, Figure 6b), suggesting improved short-term memory after CTX administration. Long-term memory was estimated based on the results averaged across four trials per day for 3 days. Both groups exhibited similar learning behaviors, and a repeated-measures ANOVA showed no significant effect of the treatment on spatial memory in the Morris water maze (F_2,17_ = 2.76, *p* = 0.09, Figure 6C).

## 3. Discussion

In this study, 5-day CTX administration led to marked LTP attenuation in CA3-CA1 synapses of young rats. LTP attenuation was associated with weakening of NMDAR-dependent signaling in hippocampal synapses. We attribute this weakening mainly to enhanced glutamate reuptake and not to changes in the expression levels of the glutamate receptors, as the PCR analysis did not show downregulation of NMDAR subunits. It is worth noting that significant LTP attenuation was not accompanied by any noticeable behavioral changes in the rats, with the behavioral tests revealing no abnormalities in the CTX-treated animals in either the open field test or Morris water maze.

The role of CTX treatment in enhancing the expression and activity of GLT1 is now well known. Since ’Rothstein’s original work [13], many studies have reported increased expression of GLT1 at both mRNA and protein levels [12,24]. Research has also demonstrated the ability of CTX treatment to elevate or normalize GLT1 expression levels in animal models of different disorders with decreased GLT1 expression [25]. In previous works, upregulation of GLT1 expression decreased the glutamate concentration in the synaptic cleft and had a neuroprotective effect [18,26]. It was also beneficial in epilepsy, stroke, hypoxia, and neurodegeneration, pointing to the potential role of GLT1 as a pharmacological target for treating these disorders [5,9,11]. CTX treatment also reduced behavioral impairment in multiple diseases. For example, 14-day CTX (200 mg/kg) administration prevented alterations in working memory in T-maze and new object recognition tests in an MPTP rat model of ’Parkinson’s disease [27]. In addition, 5-day CTX treatment normalized impaired spatial memory in rats with chronic cerebral hypoperfusion [28]. Impaired LTP in the dentate gyrus, reduced dendritic spines, and memory dysfunction in a fear conditioning test were observed in aquaporin-4 knockout mice with a decreased expression level of GLT1, all of which were rescued by 7-day CTX administration (200 mg/kg) [29].

**Figure 7:**
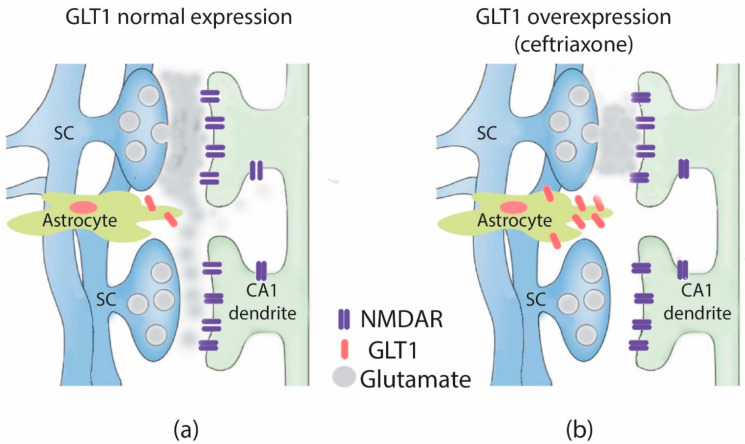
A scheme illustrating the attenuation of the glutamate spillover with GLT1 expression increase. In control, glutamate released from neighboring terminals might pool and spill over to adjoining synapses and extrasynaptic receptors (**a**), while GLT1 overexpression attenuates the glutamate spillover (**b**). For simplicity, only NMDA receptors are shown. SC-Schaffer collateral, GLT1-glutamate transporter 1.

The effects of GLT1 overexpression in control animals on synaptic transmission, synaptic plasticity, and cognitive function have been studied less thoroughly. We can assume that the impact of GLT1 overexpression on hippocampal synaptic transmission will be more pronounced under intensive stimulation, e.g., during LTP-inducing stimulation protocols, due to the specific morphological features of these synapses. For example, astrocytic processes contact less than half of Schaffer collateral-CA1 synapses [30]. Therefore, glutamate released from neighboring terminals might pool and spill over to adjoining synapses and extrasynaptic receptors [31]. According to previous studies, glutamate-mediated cross-talk is likely to be particularly important for 30–50% of hippocampal synapses [32,33]. It should also be noted that simulations predict that only NMDARs but not AMPARs localized at neighboring synapses are likely to be activated [32]. In contrast, an increase in GLT1 expression diminishes spillover and cross-talk between neighboring synapses due to more effective buffering and removal of glutamate [33] (Figure 7). For example, a postembedding immunogold study performed at MF–CA3 synapses demonstrated an increased density of membrane-associated GLT1 [20]. Its presence closer or within glutamate release sites indicates that glutamate clearance is more efficient in CTX-treated rats [20].

In line with these data, the present results revealed decreased NMDAR-dependent currents during the train stimulation in the CTX-treated animals. The reduced activation of NMDARs can likely explain the difference in the effect of MK-801 on synaptic plasticity between HFS and TBS protocols. Since MK-801 is an activity-dependent channel blocker, NMDARs activation is necessary for its effective action. However, since the TBS protocol causes only partial activation of NMDARs, the blocking efficacy of MK-801 is reduced, and therefore no effect of MK-801 on LTP was detected.

In our previous study, using the same model, we detected no difference in the properties of AMPAR-mediated eEPSP trains in control and CTX-treated rats [14]. The data from the PCR analysis in the present study support the assumption of spillover reduction. Despite the decrease in the amplitude of NMDAR-mediated currents during burst stimulation, the expression level of the obligate GluN1 receptor subunit did not change. Therefore, the reduced amplitude of the train response can be attributed to a decrease in the number of involved neighboring synapses and extrasynaptic NMDARs.

In addition, the PCR analysis pointed to increased expression of GluN2b-containing NMDARs, which are characterized by longer tau decay [1,34]. Interestingly, in our electrophysiological experiments, we found a tendency toward decreased tau decay, which does not agree with the data from the PCR analysis. However, the kinetics of NMDARs depend not only on their subunit composition but also on the activity of glutamate transporters [35]. Thus, GLT upregulation may have a marked influence on response kinetics in CTX-treated animals.

As reported previously, LTP attenuation might be directly attributed to a decrease in NMDA-dependent signaling during LTP induction protocols, as LTP in CA3-CA1 synapses is an NMDAR-dependent process [36]. However, the mechanism of LTP attenuation may be more complex and depend on the spatial arrangement of NMDARs with different subunit compositions. Numerous studies have shown that NMDARs with different subunit compositions are unevenly distributed and have different functions (for review see [37]). While GluN2a-containing NMDARs are localized mainly at synaptic sites, GluN2b-containing NMDARs are relatively more common peri-synaptically or extrasynaptically [37,38]. In addition, GluN2b-containing NMDARs exhibit higher sensitivity to glutamate than GluN2a-containing NMDARs [1,39,40]. Therefore, an increase in GLT1 activity can affect the activity of NMDARs with different subunit compositions during the LTP induction protocol to varying degrees.

In terms of synaptic plasticity, several lines of evidence point to differential roles of GluN2A and GluN2B subunits in triggering LTP and LTD. In previous research, LTP decreased in GluN2A knockout mice at CA3-CA1 synapses [41], whereas GluN2B loss abolished LTD [42]. Ifenprodil, a selective GluN2B antagonist, specifically inhibited LTD, and NVP-AAM077, a GluN2A antagonist, suppressed LTP but not LTD [43]. However, this idea is an over-simplification, as other studies have shown that GluN2A and GluN2B subunits play significant roles in LTP and LTD [43,44,45]. Xu et al. (2009) suggested that the production ratio of GluN2A and GluN2B subunits is more critical than either subunit alone in determining the sign of synaptic plasticity (LTP vs. LTD) [46]. Thus, changes in metaplasticity can also be assumed [39].

It should be noted that we examined synaptic plasticity the day after the administration of CTX for 5 days. Therefore, we do not know how long the observed decrease in LTP may continue. A previous study indicated that increased GLT1 expression may be observed for up to 3 months after CTX treatment [13]. Therefore, long-term disturbances in synaptic plasticity due to CTX cannot be ruled out.

In addition, it is important to note that we did not detect any obvious behavioral abnormalities in our study, with the behavior of the CTX-treated rats in the open field test and Morris water maze no different from that of the control animals, except for the rate of failed trials in the maze during the first day of learning.

Only a few published studies have estimated the impact of CTX treatment on rodent behavior. In one study, 9-day administration of CTX (200 mg/kg) did not alter either the distance traveled to the platform during the learning phase or the time spent in the target quadrant in the Morris water maze, despite a significant increase in GLT1 expression in CA1 and CA3 synapses and the dentate gyrus in mice [47]. However, another study demonstrated impaired memory in rats in a new object recognition test after CTX administration for 8 days [19]. It should also be noted that CTX treatment can restore hippocampus-dependent behavioral disorders in various models of disease. For example, learning and memory ability in the Morris water maze was rescued by CTX administration during the phase of epileptogenesis in the lithium-pilocarpine model of temporal lobe epilepsy [48]. Administration of CTX for 7 days and 14 days during hypoxic exposure ameliorated hypobaric hypoxia-induced cognitive impairment in the Morris water maze and GLT1 expression [49]. Therefore, the effect of CTX treatment on memory function also depends on the expression level of GLT1.

Overall, our data provide further evidence that synaptic plasticity is critically dependent on NMDAR-mediated signaling. Changes in NMDAR-mediated signaling due to GLT1 gain-of-function lead to impaired plasticity. The ability of CTX to enhance the expression and functional activity of GLT1 may likely negatively affect learning in some cases. This phenomenon can be considered a potential side effect of CTX treatment.

## 4. Materials and Methods

### 4.1. Animals and CTX Administration

The experiments were carried out on three-week-old male Wistar rats. Rats were bred at the animal facility of the Sechenov Institute of Evolutionary Physiology and Biochemistry of the Russian Academy of Sciences (Saint Petersburg, Russia). The animals were kept under standard conditions with free access to food and water. All animal procedures followed the guidelines of the European Community Council Directive 86/609/EEC and the guidelines on the treatment of laboratory animals of the Sechenov Institute of Evolutionary Physiology and Biochemistry. CTX (200 mg/kg, i.p.) was freshly dissolved in sterile saline immediately before administration. The drugs were administered at an injection volume of 2 mL/kg/day for five days.

### 4.2. Slice Preparation

Rats were sacrificed via decapitation, and their brains were removed rapidly. The brain slice preparation was described previously [23]. Horizontal 350–400-μm-thick slices were cut with a vibrating microtome (Microm HM 650 V; Microm, Walldorf, Germany). Artificial cerebrospinal fluid (ACSF) with the following composition (in mM) was used: 126 NaCl, 24 NaHCO_3_, 2.5 KCl, 2 CaCl_2_, 1.25 NaH_2_PO_4_, 1 MgSO_4_, and 10 dextrose. The ACSF was aerated with the gas mixture of 95% O_2_ and 5% CO_2_. The slices were then transferred to oxygenated ACSF and incubated for one hour at 35 °C before electrophysiological recordings. One to five slices from each rat were used in the experiment.

### 4.3. Field Potential Recordings

After incubation, the slices were placed in a recording chamber and perfused with a constant flow of ACSF at a rate of 5–7 mL/min. The electrophysiological experiment began 15–20 min after placing the slices in a chamber. Field excitatory postsynaptic potentials (fEPSPs) were recorded from CA1 stratum radiatum using glass microelectrodes (0.2–1.0 MΩ) filled with ACSF. Synaptic responses were evoked by local extracellular stimulation of afferent fibers using tungsten bipolar electrodes placed in the stratum radiatum at the CA1–CA2 border, approximately 1 mm from the recording electrode [50]. Test stimuli were delivered as constant voltage rectangular paired pulses (duration, 0.1 ms; interstimulus interval, 50 ms) every 20 s via an A365 stimulus isolator (World Precision Instruments, Sarasota, FL, USA). Responses were amplified using a Model 1800 amplifier (A-M Systems, Carlsborg, WA, USA) and digitized and recorded to a personal computer using ADC/DAC NI USB-6211 (National Instruments, Austin, TX, USA) and WinWCP v5.x.x software (University of Strathclyde, Glasgow, UK). The electrophysiological data were analyzed using Clampfit 10.2 program (Axon Instruments, San Jose, CA, USA). The amplitude and slope of the rising phase at a level of 20–80% of the peak amplitude were measured.

### 4.4. LTP of Excitatory Synaptic Transmission

LTP was assessed 1 d after the last administration of CTX. The stimulation ’current’s value was adjusted to elicit a response with a magnitude of 40–50% of maximal. Once fEPSP amplitudes were stable for 20–25 min (baseline), theta-burst stimulation (TBS, 5 trains applied five times every 10 s consisting of 5 bursts of 5 100-Hz pulses; interburst interval-200 ms) or high-frequency stimulation (HFS, 3 trains of 100 pulses at 100 Hz applied every 20 s) protocols were used to induce LTP. The LTP magnitude was defined as the average slope of the fEPSP 48–60 min after the induction protocols normalized to the slope’s mean value for the 10-min period immediately before the protocol.

### 4.5. Patch-Clamp Recordings

Whole-cell patch-clamp recordings were performed using the cesium-methanesulfonate-based pipette solution (in mM: 127 cesium methanesulfonate (CsMeSO_4_), 10 NaCl, 5 ethylene glycol-bis (β-aminoethyl ether)-N,N,N 0,N 0-tetraacetic acid (EGTA), 10 4-(2-hydroxyethyl)-1-piperazineethanesulfonic acid (HEPES), 6 QX314, 4 ATP-Mg, and 0.3 GTP, pH 7.25, adjusted with CsOH). No correction for series resistance was applied. The EPSCs were elicited by applying electrical stumuli to Schaffer collateral axons with the bipolar woven wolfram wire electrode. Repeated recordings were acquired with 20 s intervals.

### 4.6. Drugs

MK-801 (10 μM), an uncompetitive NMDAR antagonist, QX314 (6 µM), voltage-gated Na^+^ channel blocker, bicuculline (10 µM), a competitive GABAa receptor antagonist, CGP 55845, a selective GABAb receptor antagonist (5 µM), and DNQX (20 µM), a competitive AMPA and kainate receptor antagonist, were obtained from Sigma (St. Louis, MO, USA). These drugs, used for the electrophysiology experiments, were diluted in distilled water and applied via a bath.

### 4.7. Quantitative PCR (qRT-PCR)

Rats were decapitated 24 h after the last injection of CTX or saline. Brains were quickly isolated, frozen, and stored at −80 °C until dissection. The dorsal hippocampus was dissected using a cryostat-microtome OTF5000 (Bright Instrument, Huntingdon, UK) according to the rat brain atlas [51]. Total RNA was isolated using the acid guanidinium thiocyanate-phenol-chloroform extraction method [52] with ExtractRNA reagent (Evrogen, Moscow, Russia) according to the ’manufacturer’s instructions. RNA concentration was assessed spectrophotometrically using NanoDrop™ Lite (Thermo Fisher Scientific, Wilmington, DE, USA) at 260 nm.

Total RNA (2 μg), oligo-dT-primers (0.5 µg per 1µg RNA), (DNA Synthesis Ltd., Moscow, Russia) and MMLV reverse transcriptase (100 units per 1 µg RNA; Evrogen, Moscow, Russia) were used for reverse transcription. The reaction was conducted in a total volume of 20 µL: we mixed primers and 8 µL of RNA solution and incubated 10 min at 70 °C and quickly cooled to 4 °C for primer annealing. Then we added reverse transcriptase-containing reaction mix, and samples were incubated 2 h at 42 °C and 10 min at 65 °C. After this step, all samples were diluted 10-fold before the PCR step.

qPCR was performed in a total volume of 10 µL with 0.8 µL cDNA, 0.75 units of TaqM-polymerase (Alkor Bio, St. Petersburg, Russia), 3.5 mM Mg^2+^, specific forward, reverse primers, and hydrolysis (TaqMan) probes (see Table 1). Nucleotides were synthesized by DNA Synthesis Ltd. (Moscow, Russia). PCR reactions were performed in a C1000 Touch thermal cycler combined with a CFX96 Touch™ Real-Time PCR Detection System (BioRad, Hercules, CA, USA) in triplets, simultaneously with no template and no reverse transcription control samples. To determine the relative expression of genes *Grin1*, *Grin2a*, *Grin2b*, *Gria1*, and *Gria2*, method 2^−ΔΔCt^ was used [53]. *Actb* and *Gapdh* were used as reference genes. Their stability in the reported experiments was confirmed in the preliminary experiments with the approach suggested earlier [54].

### 4.8. Behavioral Tests

Behavioral tests started the next day after the last CTX (CTX group, *n* = 10) or saline (Control group, *n* = 10) administration. The open field test was performed to evaluate the locomotor activity and the anxiety level. Rats were placed into a round arena with a diameter of 1 m, the height of the wall of 30 cm, and a lightness of 8 Lx. Rat movements were video recorded, and tracks were analyzed software "Round and Cross" (Institute of Experimental Medicine, St. Petersburg, Russia) [60]. Distance traveled during 3 min of testing was used to estimate the locomotor activity and time spent in the center of the arena (¼ of the arena’s radius) and thigmotaxis (less than 7 cm from the wall) for estimation of the anxiety level.

Two days after the last injection of CTX, a Morris water maze test was performed to evaluate the spatial memory. The maze was a round pool with a diameter of 150 cm and walls of 70 cm height with opaque water (22 ± 1 °C). The walls were marked with four different signs as spatial landmarks. During three consecutive days, rats were taught to find the 10 × 10 cm platform located 15 cm from the wall and hidden 1 cm under the water. Each day rats had four 90-s trials with a 2-min break. During each attempt, the rat had to find the platform and then stay there for 30 s before being moved to the cage. If the rat failed to find the platform, it was directed to the platform manually. Such trial was marked as failed, and the distance passed during 90 s was taken into the analysis. Rat movements were video recorded, and tracks were analyzed with “Round and cross” software.

### 4.9. Statistics

The statistical analysis and graphical representation of the results were performed using OriginPro 8 (OriginLab Corp., Northampton, MA, USA) and Statistica 8.0 (StatSoft Inc., Tulsa, OK, USA). Dixon’s Q test (at a 90% confidence level) was used to identify and reject outliers. The normality of the sample data was evaluated using the Shapiro-Wilk test or Kolmogorov–Smirnov test. The equality of variance was assessed using the Levene median test. Statistical significance was assessed using Student’s *t*-test and ANOVA as stated in the text. All data are presented as mean ± standard error of the mean. *p* < 0.05 was considered statistically significant.

## Figures and Tables

**Table 1:** Primers and probes used in qRT-PCR.

Gene Symbol RefSeq Accession Number	Nucleotide Sequences (Forward, Reverse, TaqMan-Probe)	Reference
Actb NM_031144	TGTCACCAACTGGGACGATA	[55] (primers) [54] (probe)
	GGGGTGTTGAAGGTCTCAAA	
	FAM-CGTGTGGCCCCTGAGGAGCAC-BHQ1	
Gapdh NM_017008	TGCACCACCAACTGCTTAG	[56]
	GGATGCAGGGATGATGTTC	
	R6G-ATCACGCCACAGCTTTCCAGAGGG-BHQ2	
Grin1 NM_017010	GTTCTTCCGCTCAGGCTTTG	[57]
	AGGGAAACGTTCTGCTTCCA	
	FAM-CGGCATGCGCAAGGACAGCC-BHQ1	
Grin2a NM_012573	GCTACACACCCTGCACCAATT	[58]
	CACCTGGTAACCTTCCTCAGTGA	
	FAM-TGGTCAATGTGACTTGGGATGGCAA-BHQ1	
Grin2b NM_012574	CCCAACATGCTCTCTCCCTTAA	[58]
	CAGCTAGTCGGCTCTCTTGGTT	
	FAM-GACGCCAAACCTCTAGGCGGACAG-BHQ1	
Gria1 NM_031608	TCAGAACGCCTCAACGCC	[59]
	TGTAGTGGTACCCGATGCCA	
	ROX-TCCTGGGCCAGATCGTGAAGCTAGAAAA-BHQ1	
Gria2 NM_017261	CAGTGCATTTCGGGTAGGGA	[59]
	TGCGAAACTGTTGGCTACCT	
	FAM-TCGGAGTTCAGACTGACACCCCA-BHQ1	

## Data Availability

The data presented in this study are available on request from the corresponding author.
